# Concurrently wasted and stunted 6-59 months children admitted to the outpatient therapeutic feeding programme in Karamoja, Uganda: Prevalence, characteristics, treatment outcomes and response

**DOI:** 10.1371/journal.pone.0230480

**Published:** 2020-03-20

**Authors:** Gloria A. Odei Obeng-Amoako, Henry Wamani, Joel Conkle, Richmond Aryeetey, Joanita Nangendo, Ezekiel Mupere, Joan N. Kalyango, Mark Myatt, André Briend, Charles A. S. Karamagi

**Affiliations:** 1 School of Medicine, Clinical Epidemiology Unit, College of Health Sciences, Makerere University, Kampala, Uganda; 2 Department of Community Health and Behavioural Sciences, School of Public Health, College of Health Sciences, Makerere University, Kampala, Uganda; 3 Health and Nutrition Section, UNICEF Namibia, Windhoek, Namibia; 4 School of Public Health, University of Ghana, Legon, Ghana; 5 Department of Paediatrics and Child Health, College of Health Sciences, Makerere University, Kampala, Uganda; 6 Department of Pharmacy, College of Health Sciences, Makerere University, Kampala, Uganda; 7 Brixton Health, Wales, United Kingdom; 8 School of Medicine, Centre for Child Health Research, University of Tampere, Tampere, Finland; 9 Department of Nutrition, Exercise and Sports, University of Copenhagen, Copenhagen, Denmark; Institut de recherche pour le developpement, FRANCE

## Abstract

This study assessed the prevalence of concurrently wasted and stunted (WaSt) children, their characteristics, treatment outcomes and response; and factors associated with time to recovery among children aged 6–59 months admitted to Outpatient Therapeutic Care (OTC) in Karamoja, Uganda. We conducted a retrospective cohort study with data from January 2016 to October 2017 for children admitted to nine OTCs in Karamoja. We defined wasted, stunted and underweight as 2.0 Z-scores below the median per WHO growth standards and < 12.5 cm for low Mid-Upper Arm Circumference (MUAC). WaSt was defined as concurrently wasted and stunted. Out of 788 eligible children included in the analysis; 48.7% (95% CI; 45.2–52.2) had WaSt. WaSt was common among males; 56.3% (95% CI; 51.3–61.3). Median age was 18 months in WaSt versus 12 months in non-WaSt children (p < 0.001). All WaSt children were underweight; and more severely wasted than non-WaSt children. During recovery, WaSt children gained weight more rapidly than non-WaSt children (2.2g/kg/day vs. 1.7g/kg/day). WaSt children had lower recovery rate (58.0% vs. 65.4%; p = 0.037). The difference in median time of recovery between WaSt and non-WaSt children (63 days vs. 56 days; p = 0.465) was not significant. Factors associated with time to recovery were children aged 24–59 months (aHR = 1.30; 95% CI;1.07–1.57;), children with MUAC 10.5–11.4 cm (aHR = 2.03; 95% CI; 1.55–2.66), MUAC ≥ 11.5 cm at admission (aHR = 3.31; 95% CI; 2.17–5.02) and living in Moroto (aHR = 3.34; 95% CI; 2.60–4.30) and Nakapiripirit (aHR = 1.95; 95% CI; 1.51–2.53) districts. The magnitude of children with WaSt in OTC shows that existing therapeutic feeding protocols could be used to detect and treat WaSt children. Further research is needed to identify and address the factors associated with sub-optimal recovery in WaSt children for effective OTC programming in Karamoja.

## Introduction

Severe Acute Malnutrition (SAM) in children under 5 years is a global public health concern. Globally, about 52 million children under 5 years are wasted and 17 million are severely wasted [1]. Nearly 4.3 million of all severely wasted children are in Africa [1]. In Uganda, 4% of children under 5 years are wasted and 1.3% are severely wasted [2].

Annually, 3.1 million of all deaths in children under 5 years are associated with under-nutrition [3]. SAM, the deadliest form of under-nutrition is known to directly cause 4.4% of child deaths in the absence of effective treatment [3]. SAM in children aged 6–59 months is defined by weight-for-height Z-scores (WHZ) < -3 of the World Health Organization (WHO)’s Child Growth Standard or mid-upper arm circumference (MUAC) < 11.5 cm and/or the presence of bilateral pitting edema [4]. This case definition forms the basis for therapeutic feeding programme admissions. The current therapeutic feeding protocol is proven to effectively treat children with SAM [5, 6]. In recent times, the need for evidence on the current therapeutic feeding programmes to detect and treat children having concurrent wasting and stunting; a condition termed WaSt has been raised [7–9]. Until now the focus of therapeutic feeding programme has been on SAM, but there is a need for further research for integrated programme approaches for addressing wasting and stunting in children [10].

Literature suggests that wasting retards linear growth leading to stunting; thus a wasted child is often stunted [11, 12]. Risk factors associated with wasting and stunting are often seen in the same child [11, 13]. Reports show children younger than 30 months especially males are mostly affected by WaSt [8, 9, 14]. The risk of death associated with severe wasting is 12 fold compared with well-nourished children [15, 16]. Children with severe stunting have about 5 times the risk of death compared with children without anthropometric deficits [15]. Children with WaSt are 12 times more likely to die compared to well-nourished children [8, 16]. This sharply contrasts with the relatively smaller risk of being either wasted (2.3 hazard ratio) or stunted alone (1.5 hazard ratio) [8, 16]. Children with WaSt have a risk of death comparable to children with severe wasting [8, 16]. The increased risk of death associated with WaSt is because of the negative synergistic effect of concurrent wasting and stunting [8].

Given the emerging evidence on WaSt in children, the basis of the current separation in programming, research and funding need to be re-examined [7, 17]. Evidence is needed on how to optimize the current therapeutic feeding protocols to detect, treat and reduce mortality associated with WaSt [8]. Also, because some children with WaSt are classified as SAM in the current therapeutic feeding programme, their outcomes and response to treatment are unknown.

We assessed the prevalence of WaSt; the characteristics, treatment outcomes and response of children with WaSt; and factors associated with time to recovery among children aged 6–59 months admitted for SAM treatment in Outpatient Therapeutic Care (OTC) in Karamoja, in North-Eastern Uganda.

## Methods

### Operational definitions

We defined wasting, stunting, and underweight by the Z-scores of 2006 WHO growth standards as follows [18].

Wasted: weight for height Z-scores (WHZ) < -2.0

Stunted: height for age Z-scores (HAZ) < -2.0

Underweight: weight for age Z-scores (WAZ) < -2.0

Degrees of anthropometric deficits were defined as: no deficit ≥-2; moderate ≥ -3 to -2 and severe < -3

WaSt: concurrently WHZ < -2.0 and HAZ < -2.0 [8].

Non-WaSt: WHZ ≥-2.0; or HAZ ≥ -2.0; or WHZ < -2.0 and HAZ ≥ -2.0; or HAZ < -2.0 and WHZ ≥-2.0; or WHZ ≥-2.0 and HAZ ≥ -2.0

Acute malnutrition was defined as wasting (WHZ<-2) and/or MUAC<12.5; severe acute malnutrition (SAM) as severe wasting (WHZ < -3) and/or MUAC < 11.5 cm and moderate acute malnutrition (MAM) as moderate wasting (WHZ ≥ -3 to -2) and/or MUAC ≥ 11.5 cm and ≤ 12.5 cm [19].

We used the following formula to define treatment response:

- Length of stay (days) as the difference between the date at discharge and date at admission;
- Weight gain (Kg) as the difference between the last recorded weight at discharge and weight at admission;
- Weight velocity (g/kg/day) = 1000 × (last recorded weight at discharge—weight at admission)/ (last recorded weight at discharge × length of stay (days));
- Proportional weight-gain (%) = (last recorded weight at discharge—weight at admission)/weight at admission) × 100;
- MUAC gain (cm) as the difference between last recorded MUAC (at discharge) and MUAC at admission [6].

### Study design and source of the dataset

This was a retrospective cohort study using a dataset consisting of treatment records of children aged 6–59 months admitted to nine OTC sites in Karamoja from January 2016 to October 2017. All the children in the dataset used for this analysis were children with SAM. The dataset was collected as part of a SAM incidence and post-treatment relapse feasibility study conducted from September to October 2017 in Karamoja (unpublished report). Treatment records were extracted from three outpatient health facilities in each of the three districts; Kaabong, Moroto, and Nakapiripirit. The purpose was to ascertain the feasibility of a proposed study on post-treatment relapse to SAM. These health facilities were purposively sampled based on their high caseloads and because they had treatment records. Seven of the nine health facilities were operated by the Government of Uganda Ministry of Health and the other two were managed as private-non-for profit/mission facilities offering OTC services.

The SAM incidence and post-treatment relapse feasibility study were carried out in collaboration with the United Nations Children's Fund (UNICEF) Uganda, Doctors with Africa (CUAMM), Ministry of Health (MoH) and local government authority in the selected districts. Before data collection, stakeholder consultations were held as part of the community entry process. Health facility data were made available to the study team by the MoH and CUAMM in the study districts.

Data on treatment records were extracted from the MoH’s Integrated Nutrition Registers (INR) in all health facilities. The INR is managed and maintained by the health workers at the health facilities. A data extraction form designed using Open Data Kit (ODK) was used to extract data from one health facility while paper-based forms were used in the remaining health facilities (n = 8). Data extraction was carried out by two trained research assistants. A total of 1345 treatment records (1181 paper-based and 164 ODK-based forms) were retrieved from the INR. The paper-based data-extraction forms were entered once into Epi-data software with in-built interactive ranges and legal value checks. Samples of the data entered were randomly checked for completeness. The two datasets were uploaded and merged into a single dataset in STATA version 13.0.

### Study setting and programme context

Karamoja is a semi-arid agro-pastoral setting in North-Eastern Uganda and has seven administrative districts: Moroto, Napak, Amudat, Nakapiripirit, Kotido, Kaabong, and Abim. Karamoja is characterized by chronic food insecurity with about half of its 1.4 million population classified as food insecure [20]. Childhood under-nutrition remains a significant public health problem. In Karamoja, 33% of children under 5 years are stunted and 28% are underweight [21]. About 90% of the health facilities (114 OTC- and 10 Inpatient Therapeutic Care (ITC) sites) in Karamoja provide SAM treatment. Technical assistance for SAM treatment services is provided by CUAMM in partnership with the MoH in Uganda Ministry of Health and UNICEF Uganda.

SAM treatment services follow the Ugandan National Integrated Management of Acute Malnutrition (IMAM) guidelines which consist of community mobilization, OTC, ITC and Supplementary Feeding Programmes (SFP). SAM treatment services are offered in public and private health facilities. Village Health Teams search for, refer and follow-up cases through community mobilization [22]. Children are screened for SAM using MUAC < 11.5 cm and bilateral edema by community health workers and health volunteers at the community level [22]. Children with MUAC < 11.5 cm or any degree of bilateral edema, are referred for a full assessment at health facilities [22]. At the health facilities, health-care workers assess MUAC and/or WHZ or bilateral pitting edema to confirm admission. Children aged 6–59 months fulfilling any of the admission criteria without medical complications and have appetite are admitted to OTC. Children with SAM plus medical complications are first treated in the ITC until complications resolve and are then transferred to OTC for further treatment. At the OTC, children receive take-home ration of Ready to Use Therapeutic Food (RUTF). Children are presented at the OTC centres by their caregivers for weekly monitoring and RUTF ration for a period of 6–8 weeks until cured and discharged. Children with MAM, are treated at the SFP centres [22].

The recommended criteria for discharge from OTC are WHZ ≥ -2 or MUAC ≥ 12.5 cm with no edema for 2 weeks [22]. However, because SFP are implemented in Karamoja, children are discharged as cured and are transferred to SFP with MUAC ≥ 11.5 cm and ≤ 12.5 cm or WHZ ≥ -3 and < -2 for further rehabilitation using Corn Soy Blend++ (CSB). Children who attain recovery from SAM to normal (WHZ ≥ -2 or MUAC ≥ 12.5 cm) are discharged home from the therapeutic feeding programme A child is classified as non-response if the discharge criterion is not fulfilled after 16 weeks of treatment. A default is defined as absence from treatment for three consecutive visits. A child whose nutritional status deteriorates in OTC is referred to or transferred to ITC [22]. For ease of analysis, recovery from SAM to MAM was used as cured in this study.

### Study population

Children who met the admission criteria of WHZ < -3 and/or MUAC < 11.5 cm, and had data on weight, height, MUAC at admission and exit with treatment outcomes were included in the analysis. We excluded children with aberrant anthropometric records and those with bilateral pitting edema because of their unusual weight gain pattern as edema diminishes during SAM treatment [23]. We also excluded children with HIV given their atypical health conditions.

### Data analysis

We used STATA software version 13.0 (StataCorp, College Station, TX, USA) and Microsoft Excel for all the statistical analyses. Z-scores above the upper quartile +1.5 × interquartile range (IQR) were censored as outliers instead of the WHO flagging criteria. Given that this analysis consisted of very sick and undernourished children, children with extreme negative Z-scores below the WHO flagging cut-off points were not censored from the study [24]. Children aged 24 months and above with extreme values for height (i.e. > 120 cm) as well as those less than 24 months with length values > 100 cm were censored. Additionally, children with MUAC > 20 cm were also censored [22].

We used a Venn diagram to assess the overlap between sets of children with nutritional deficits [8, 25]. We described the prevalence of WaSt versus non-WaSt and their characteristics (age, sex, baseline weight, height, Z-scores, and MUAC) with summary statistics. We summarized continuous variables using medians, interquartile ranges (IQRs) and percentages for categorical variables. We compared treatment outcomes and responses in two groups; WaSt and non-WaSt children using Chi-square (χ^2^) for categorical variables and Wilcoxon rank-sum test for continuous variables.

We estimated and compared the median time to recovery by groups; sex, age categorized 6–23 months and 24–59 months, WaSt, and geographical location using the Kaplan Meier survival method and the Log Rank test. We used multivariate Cox proportional hazard regression analysis to assess factors associated with time to recovery. The outcome of interest was time to recovery (event), i.e. being discharged as cured and had attained MUAC ≥ 11.5 cm and ≤ 12.5 cm or WHZ ≥ -3 and < -2. All children who had not recovered (i.e. died, defaulted, non-response, and transferred to OTC/ITC) during treatment as well as those discharged from treatment as cured but had not attained nutritional recovery were classified as non-recovered and censored. We considered sex, age categorized at 6–23 months and 24–59 months and MUAC categorized into MUAC < 10.5 cm, MUAC 10.5–11.4 cm, MUAC ≥ 11.5 cm, WaSt and geographical location of children in the Cox proportional hazard regression model. Variables with p < 0.2 found in the bivariate regression analysis were included in the multivariate analysis. Factors associated with time to recovery were reported by adjusted Hazard Ratio (aHR) at 95% Confidence Interval (CI). We set statistical significance at a 95% Confidence Interval (CI) and p < 0.05 in this analysis.

### Ethics

Permission and ethical approval to conduct the SAM incidence and post-treatment relapse feasibility study was granted by the Clinical Epidemiology Unit and the School of Medicine Higher Degrees Research and Ethics Committee respectively. Ethical approval to conduct the current study was obtained from the Makerere University School of Medicine Higher Degrees Research and Ethics Committee and the Uganda National Council for Science and Technology. All treatment records of children were anonymized before we accessed them in the dataset. A waiver of consent from Makerere University School of Medicine Higher Degrees Research and Ethics Committee was sought to use this dataset for the study.

## Results

### Study profile

**Fig 1:**
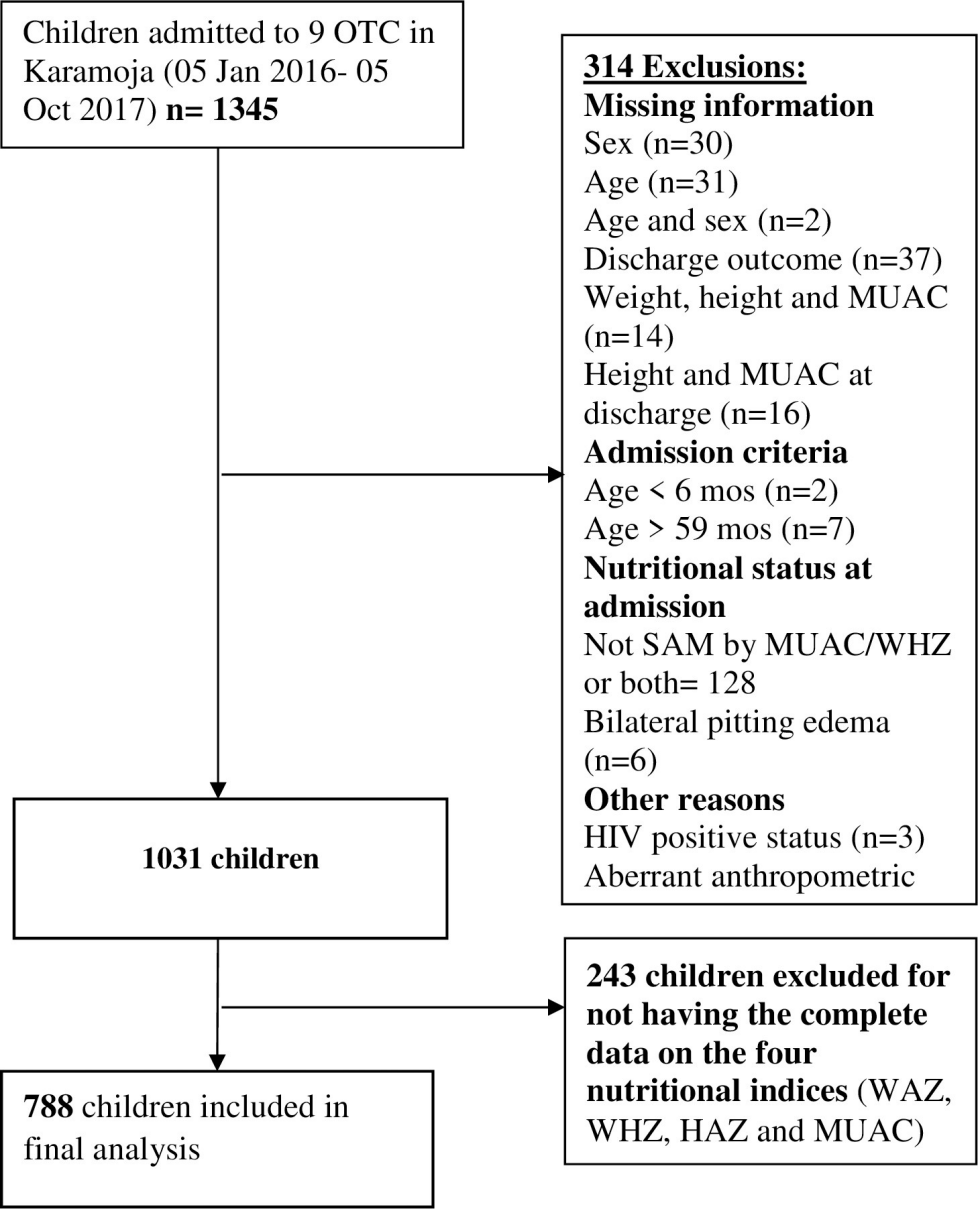
Flow chart showing participant selection among children 6–59 months admitted to OTC in Karamoja.

A total of 1345 treatment records of children treated for uncomplicated SAM from January 2016 to October 2017 were retrieved from nine health facilities in Karamoja. The flow chart (Fig 1) shows how eligible children were considered in our analysis. Seven hundred eighty-eight children with complete data on the four anthropometric indices; underweight, wasted, stunted and low MUAC in the sample were included in the final analysis. Complete data on the four anthropometric indices facilitated analysis on overlapping deficits. About half of our sample were females (52.3%) and the median age was 15 months (IQR; 10,12). There was no significant difference in the sex (males; 48.5% vs. 47.7%; p = 0.210) and age distribution (median 15 months vs. 15 months; p = 0.394) between the children in our sample and excluded children.

## Nutritional status at admission

Of the 788 children in the study sample, 99.4% (n = 783) had low MUAC and 93.1% (n = 734) were underweight (Table 1).

**Table 1:** Nutritional status at admission in children admitted to OTC, Karamoja.

N = 788	WAZ	WHZ	HAZ	MUAC
Nutritional status^a^	n (%)	n (%)	n (%)	n (%)
No deficit	54 (6.9)	164 (20.8)	279 (35.4)	5 (0.6)
Moderate	161 (20.4)	228 (28.9)	169 (21.4)	48 (6.1)
Severe	573 (72.7)	396 (50.3)	340 (43.2)	735 (93.3)

^a^WAZ: underweight; WHZ: wasted; HAZ: stunted, MUAC: mid-upper arm circumference.

### Overlap of nutritional deficits

**Fig 2:**
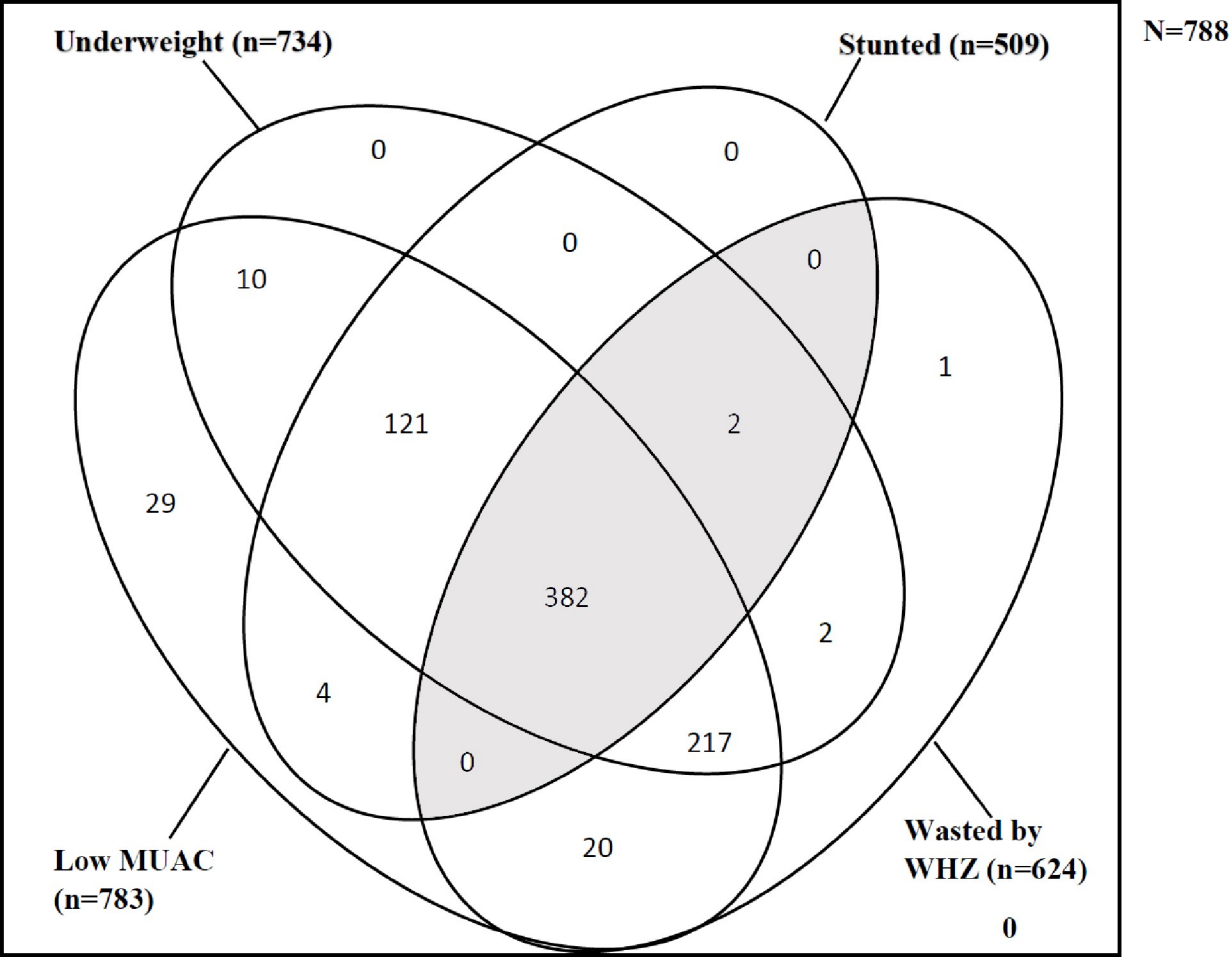
Venn diagram showing wasting, stunting, underweight and low MUAC overlaps in the study sample (N = 788). Legend: The gray shaded areas depict intersection between wasting and stunting; WaSt.

Almost half (48.7%, 384/788) of the children were WaSt (Fig 2). All four nutritional deficits overlapped in virtually all the children with WaSt (382/384). The shaded portions in the Venn diagram show the intersection of children with stunting and wasting (Fig 2). The Venn diagram also shows that all WaSt children were underweight. There was a substantial degree of overlap between underweight and low MUAC, and thus 92.6% were underweight and had low MUAC concurrently. There was no child without a nutritional deficit in the Venn diagram given that the cohort was children with SAM (Fig 2).

### Prevalence, age and sex patterns of WaSt among children admitted to OTC

**Fig 3:**
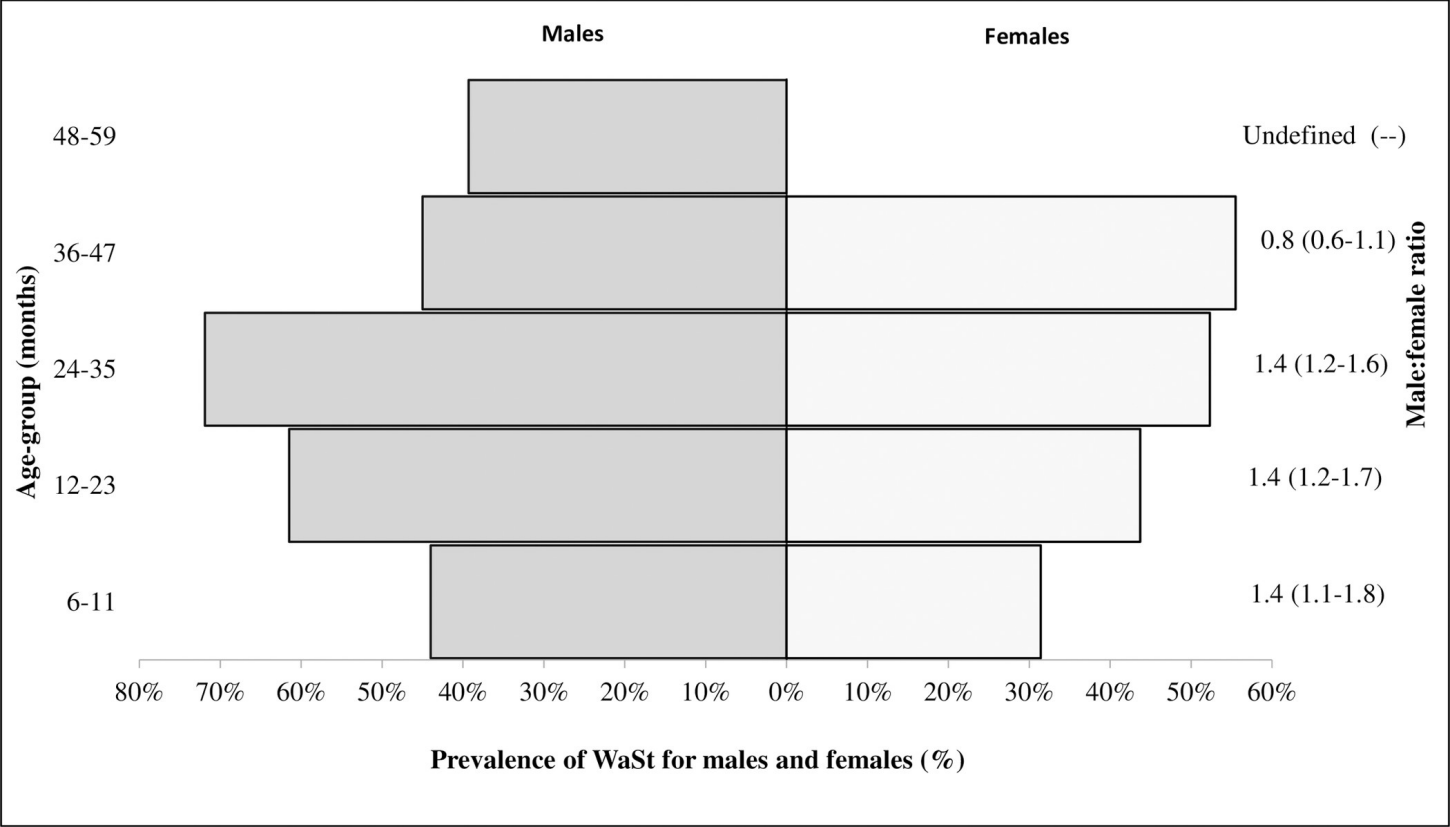
Prevalence of WaSt by age group and sex among children admitted to OTC in Karamoja.

About half of the children (48.7%; 95% CI: 45.2–52.2) admitted to OTC had WaSt. Overall, our sample had more females than males (male: female ratio = 0.91) whereas among WaSt children male to female ratio was 1.35 (95% CI; 1.17–1.56) (Tables 2 and 3). WaSt was more frequent among male children aged 6–35 months and among female children aged 36–47 months (Fig 3). WaSt peaked at 24–35 months (71.9%) in males but at 36–47 months (55.6%) in females (Fig 3).

**Table 2:** Prevalence of WaSt among children admitted to OTC, Karamoja.

Attribute	Total (n)	WaSt n (%)	95% CI^a^
**Overall**	788	384 (48.7)	45.2–52.2
**Sex**			
Male	376	212 (56.3)	51.3–61.3
Female	412	172 (41.7)	37.1–46.6
**Age groups (months)**			
6–23	499	222 (44.5)	40.2–48.9
24–59	289	162 (56.1)	50.3–61.9

^a^CI: Confidence Interval.

**Table 3:** Socio-demographic and anthropometric characteristics of WaSt versus non-WaSt children admitted to OTC, Karamoja.

Attribute	WaSt (n = 384)	Non-WaSt (n = 404)	p-value^a^
**Socio-demographic characteristics**			
Sex^b^			
Male	212 (56.4)	164 (43.6)	<0.001
Female	172 (41.7)	240 (58.3)	
Age (months)^c^			
All children	18 (12, 24)	12 (9, 24)	<0.001
Males	18 (12, 24)	14 (10, 27)	0.167
Females	16 (11, 24)	12 (9, 24)	<0.001
Age group (months)^**b**^			
6–23	222 (44.5)	277 (55.5)	0.002
24–59	162 (56.1)	127 (43.9)	
**Anthropometric measurements**^b^			
Weight (Kg)	6.3 (5.6, 7.2)	6.7 (6.0, 8.0)	<0.001
Height (cm)	70.0 (66.0, 75.0)	70.0 (65.6, 81.0)	0.021
MUAC (cm)	11.0 (10.4, 11.2)	11.0 (10.6, 11.2)	0.002
**Z-scores**^**c**^			
WAZ	-4.2 (-4.8, -3.7)	-3.0 (-3.7, -2.4)	<0.001
HAZ	-3.5 (-4.3, -2.7)	-1.6 (-2.8, -0.5)	<0.001
WHZ	-3.3 (-3.8, -2.6)	-2.6 (-3.8, -1.6)	<0.001
**MUAC categories**^**b**^			
<10.5cm	99(64.5)	57 (36.5)	<0.001
10.5–11.4 cm	262 (45.3)	317 (54.7)	
≥ 11.5cm	23 (43.4)	30 (56.6)	
**Geographical location (districts)** ^**b**^			
Kaabong	117(47.6)	129 (52.4)	0.697
Moroto	151 (50.7)	147 (49.3)	
Nakapiripirit	116 (47.5)	128 (52.5)	

^a^Proportions were compared using Chi-square (χ^2^) and continuous variables were compared using Wilcoxon rank-sum test.

^b^n (%): number (percent);

^c^Median (IQR) Interquartile Range.

### Socio-demographic and anthropometric characteristics of WaSt versus non-WaSt children

The median age of children with WaSt (18 months) and non-WaSt (12 months) was statistically different (p < 0.001). Children with WaSt had significantly lower median MUAC (p = 0.002) and were more severely underweight (median WAZ; -4.2 vs. -3.0; p < 0.001), wasted (median WHZ; -3.3 vs. -2.6; p < 0.001), and stunted (median HAZ; -3.4 vs. -1.6; p < 0.001) compared to children without WaSt. Prevalence of WaSt versus non-WaSt in the study districts were not statistically different (Kaabong; p = 0.658, Moroto; p = 0.395 and Nakapiripirit; p = 0.654) (Table 3).

### Treatment outcome and response

Children with WaSt had lower recovery rate compared with children without WaSt (58.0% vs. 65.4%; p *<* 0.037). More children with WaSt were discharged as non-responsive to treatment than non-WaSt children (18.7% vs. 9.8%; p < 0.001). WaSt children had slightly longer length of treatment (63 days vs. 56 days; p = 0.465) than non-WaSt children although the difference was not statistically significant. Children with WaSt had slightly greater weight velocity per day than non-WaSt children (2.2 g/kg/day vs.1.7 g/kg/day; p = 0.004). The median MUAC gain did not differ between groups (p = 0.514) (Tables 4 and 5).

**Table 4:** Treatment outcomes of WaSt versus non-WaSt children admitted to OTC in Karamoja.

Outcome	WaSt (n = 369)	Non-WaSt (n = 387)	p-value^a^
	n (%)	n (%)	
Recovered^b^	214 (58.0)	253 (65.4)	0.037
Transferred to OTC/ITC	9 (2.4)	3 (0.8)	0.067
Defaulted	73 (19.8)	90 (23.3)	0.246
Non response	69 (18.7)	38 (9.8)	< 0.001
Died	4 (1.1)	3 (0.8)	0.658

^a^Proportions were compared using Chi-square (χ^2^)

^b^Only children who attained MUAC ≥ 11.5 to < 12.5 cm or WHZ ≥ -3.0 to < -2.0 or both and discharged as recovered were included in the analysis; WaSt (n = 214) and non-WaSt (n = 253).

**Table 5:** Treatment response of recovered WaSt and non-WaSt children admitted to OTC, Karamoja.

Treatment response	WaSt (n = 214)	Non-WaSt (n = 253)	p-value^a^
	Median (IQR)	Median(IQR)	
Length of stay (days)	63 (36, 84)	56 (35, 91)	0.465
Weight gain (Kg) ^b^	1.0 (0.6, 1.5)	0.8 (0.5, 1.4)	0.007
Weight velocity (g/kg/day) ^b^	2.2 (1.2, 3.4)	1.7 (1.0, 2.8)	0.004
Proportional weight gain(%)^b^	14.8 (8.8, 23.2)	11.7 (6.8, 20.0)	0.001
MUAC gain (cm)^c^	1.1 (0.6, 1.6)	1.1 (0.7, 1.6)	0.514

^a^Continuous variables were compared using Wilcoxon rank-sum test.

^b^Reported for children with available weight data at discharge in the data set: WaSt (n = 214) and non-WaSt (n = 251).

^c^Reported for children with available MUAC data at discharge in the data set: WaSt (n = 203) and non-WaSt (n = 241).

### Incidence rate and median time to recovery

The overall incidence rate of recovery from SAM in the cohort was 8.0 (95% CI; 7.3–8.8) per 1000 person-days. The incidence rates of recovery were 7.4 (95% CI; 6.5–8.-5) per 1000 person-days and 8.6 (95% CI; 7.6–9.7) per 1000 person-days among WaSt and non-WaSt children respectively. Among the recovered children, WaSt children had an incidence rate of recovery of 14.5 (95% CI; 12.7–16.6) per 1000 person-days whereas non-WaSt children had an incidence rate of recovery of 14.7 (95% CI; 13.0–16.6) per 1000 person-days. There was no significant difference between the incidence rate of recovery between WaSt and non-WaSt children among the recovered children (Table 6).

**Table 6:** Incidence rates and median time to recovery by groups among children admitted to OTC, Karamoja.

Groups	Person time (Days)	Recovered (n/N)^a^	Incidence rate^b^ (95% CI)	Median time to recovery^c^ (95% CI)	Log rank (χ2)	p-value^d^
**All children**	58249	467/ 784	8.0 (7.3–8.8)	84 (77–91)		
**Sex**						
Males	29007	226/375	7.8 (6.8–8.9)	83 (77–98)	0.75	0.388
Females	29242	241/409	8.2 (7.3–9.4)	84 (70–98)		
**Age groups (months)**						
6–23	37328	276/495	7.4 (6.6–8.3)	91 (77–105)	5.07	0.024
24–59	20921	191/289	9.1(7.9–10.5)	76 (63–90)		
**WaSt**						
No	29538	253/403	8.6 (7.6–9.7)	77(70–91)	2.39	0.122
Yes	28711	214/381	7.4 (6.5–8.5)	90(77–105)		
**MUAC**						
< 10.5 cm	12202	56/153	4.6 (3.5–6.0)	119 (105–133)	25.48	<0.001
10.5–11.4 cm	42395	369/578	8.7 (7.9–9.6)	77 (70–84)		
≥ 11.5 cm	3652	42/53	11.5 (8.5–15.6)	63 (49–91)		
**Geographical location (districts)**						
Kaabong	25479	160/245	6.3 (5.4–7.3)	126 (108–147)	73.65	<0.001
Moroto	16025	181/298	11.3 (9.8–13.1)	63 (56–70)		
Nakapiripirit	16745	126/241	7.5 (6.3–10.0)	82 (75–96)		
**Incidence rates and median time to recovery among the recovered children**						
**All children**	31973	467	14.6 (13.3–16.0)	58 (56–63)		
**WaSt**						
No	17223	253	14.7(13.0–16.6)	56 (51–62)	0.02	0.898
Yes	14750	214	14.5(12.7–16.6)	63 (56–63)		

^a^n/N: number recovered out of number of the participants.

^b^Incidence rate expressed in 1000/person/days.

^c^Median time measured in days.

^d^Median time to recovery compared with Log Rank test (χ2).

**Fig 4:**
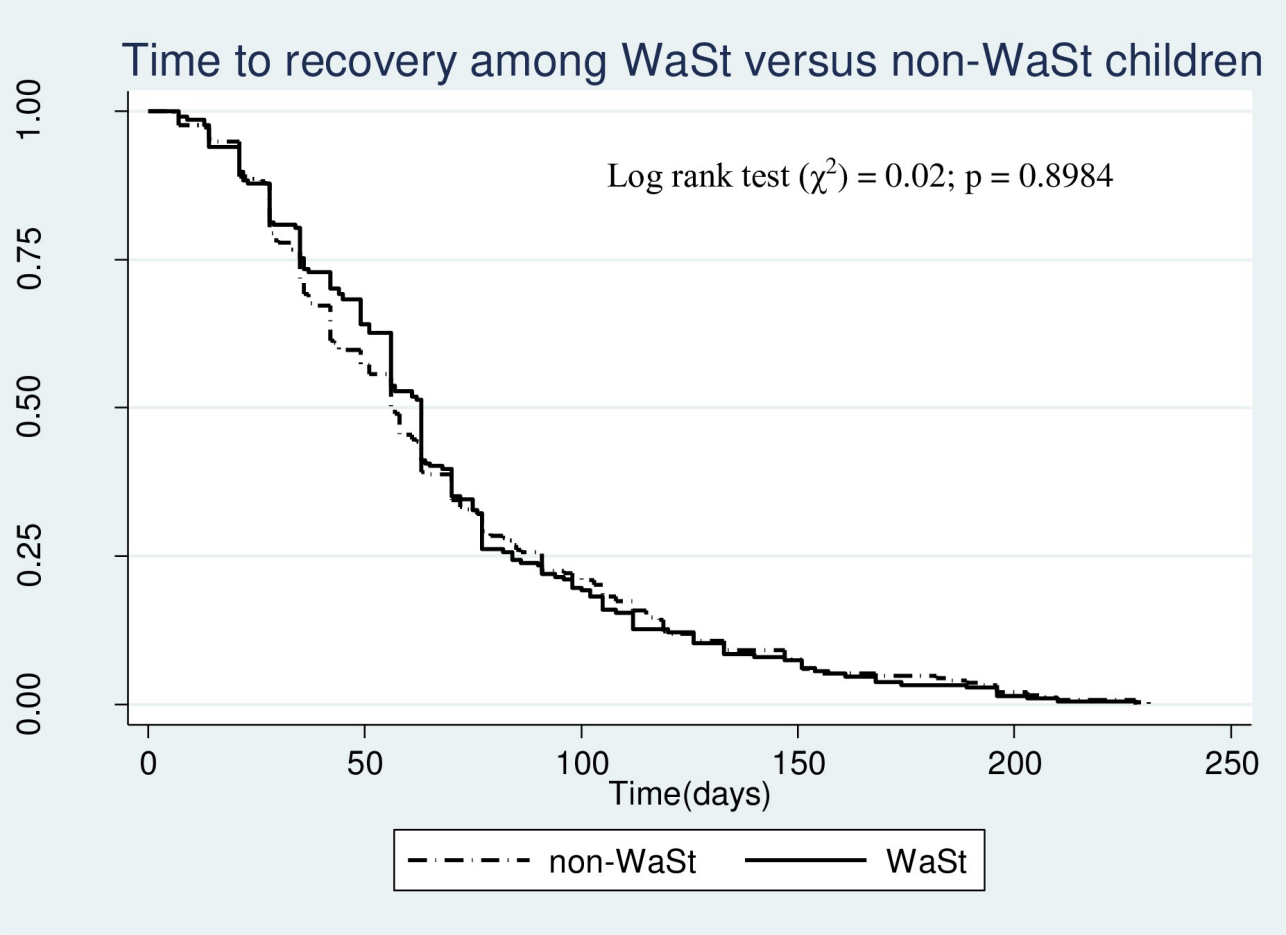
Comparison of Kaplan Meier survival curves of WaSt vs. non-WaSt children among children who recovered from SAM in OTC, Karamoja.

The overall median time to recovery in the cohort was 84 days (95% CI; 77–91). The median time to recovery was slightly longer in children with WaSt compared with children without WaSt (63 days vs. 56 days; p = 0.898) although the difference was not statistically significant (Table 6 and Fig 4).

The median time to recovery was 119 days (95% CI; 105–133), 77 days (95% CI; 70–84) and 63 days (95% CI; 49–91) for children who had MUAC < 10.5 cm, MUAC 10.5–11.4 cm and ≥ 11.5 cm at admission respectively. The difference in median time to recovery by the MUAC categories was statistically significant (p < 0.001) (Table 6).

### Factors associated with time to recovery

In the multivariate Cox proportional hazard regression analysis, females (aHR = 1.10; 95% CI; 0.92–1.32; p = 0.296) and children without WaSt (aHR = 1.20; 95% CI; 0.98–1.43; p = 0.081) had an increased probability of recovery compared with males and children with WaSt, though the differences were not statistically significant. Age, MUAC and geographical location had significant influence on time to recovery. Children aged 24–59 months (aHR = 1.30; 95% CI;1.07–1.57; p = 0.007), those with MUAC 10.5–11.4cm (aHR = 2.03; 95% CI; 1.55–2.66; p < 0.001) and MUAC ≥ 11.5cm (aHR = 3.31; 95% CI; 2.18–5.02; p < 0.001) at admission; and living in Moroto district (aHR = 3.34; 95% CI; 2.60–4.30; p < 0.001) and Nakapiripirit district (aHR = 1.95; 95% CI; 1.51–2.53; p < 0.001) had increased probability of recovery (Table 7).

**Table 7:** Multivariable analysis on factors associated with time to recovery among children admitted to OTC, Karamoja.

Attribute	Bivariate		Multivariate	
	cHR (95% CI)	p-value	aHR (95% CI)	p-value
**Sex**				
Male	1		1	
Female	1.08 (0.91–1.29)	0.383	1.10 (0.92–1.32)	0.296
**Age**				
6–23	1		1	
24–59	1.23 (1.02–1.48)	0.027	1.30 (1.07–1.57)	0.007
**WaSt**				
Yes	1		1	
No	1.15 (0.96–1.37)	0.121	1.20 (0.98–1.43)	0.081
**MUAC**				
< 10.5 cm	1		1	
10.5–11.4 cm	1.86 (1.43–2.42)	<0.001	2.03 (1.55–2.66)	<0.001
≥ 11.5 cm	2.47 (1.65–3.69)	<0.001	3.31 (2.18–5.02)	<0.001
**Geographical location**				
Kaabong district	1			
Moroto district	2.70 (2.12–3.42)	<0.001	3.34 (2.60–4.30)	<0.001
Nakapiripirit district	1.63 (1.26–2.12)	<0.001	1.95 (1.51–2.53)	<0.001

cHR and aHR stands for crude and adjusted Hazard Ratios.

## Discussion

To the best of our knowledge, our analysis on the prevalence of WaSt children, their characteristics, treatment response and outcomes; and factors associated with recovery among children admitted to OTC is novel. Earlier analyses on WaSt were based on population-based survey data [9, 14, 16, 26]. The findings of our analysis add to the existing evidence on children with WaSt.

### Prevalence of children with WaSt

Our study showed the magnitude of WaSt among children admitted to OTC. Nearly, half of the children in our sample had WaSt. Previous analysis of population-based cross-sectional data from 84 countries reported the prevalence of WaSt ranging from 0% to 8.0% and a pooled prevalence of 3.0% (95% CI; 2.97–3.06) [9]. The finding that about half of the children admitted to OTC had WaSt implies that WaSt is highly prevalent especially in high under-nutrition burden settings. These findings indicate the need to routinely monitor this condition, as well as to investigate whether children with WaSt are being reached through existing therapeutic feeding programmes [9].

### Characteristics of children with WaSt

WaSt was common in younger children aged 6–36 months in both sexes; with males more affected at 1.35 male to female ratio. Thus could indicate males are more susceptible to WaSt than females [9, 12, 14]. The risk factors associated with WaSt are not fully understood [11, 13]. However, emerging evidence shows that younger children less than 30 months and especially males are at high risk of WaSt [8, 9, 12, 14]. Furthermore, the present analysis showed WaSt children had a median age of 18 months compared with 12 months in those without WaSt. Males experienced a later onset of WaSt at 18 months compared to females at 16 months. Contrary to literature, we observed a rise in WaSt among females aged 36–47 months; largely because more females (52%) were admitted to OTC. These findings draw our attention to prioritize and improve community-based screening activities targeted at younger children especially those aged < 36 months. Future studies on WaSt prevalence, sex and age patterns using population-based data would be essential to inform screening and case detection activities.

All WaSt children were also underweight (WAZ < -2) and there was no child with stunting alone in the current analysis. The median WAZ in WaSt was as low as -4.2 (IQR; -4.8, -3.7) compared with -3.0 (IQR -3.7, -2.4) among the non-WaSt children. All the four anthropometric deficits; wasted, stunted, underweight and low MUAC overlapped in WaSt children. We observed that low MUAC and underweight overlapped in the majority (92.6%) of the study sample. This was consistent with other reports which showed that children with WaSt tend to have worse wasting and stunting Z-scores and are all underweight (WAZ < -2.0) [8, 14]. We found a range of WAZ in WaSt from a minimum of -6.83 to a maximum of -2.8. A previous report showed a maximum WAZ of -2.36 in WaSt suggesting the low WAZ in WaSt could be attributed to the joint effect of wasting and stunting [8]. This highlights the potential utility of MUAC along with WAZ for screening and detecting WaSt in children at the community and health facility levels [8]. Future research on the utility of MUAC and WAZ to detect WaSt children using population-based data is recommended. Further research on how to integrate community case finding and referral protocol for WaSt into the existing therapeutic feeding programmes would be needed.

### Treatment outcomes and response

In the present analysis, the rate of recovery from SAM to MAM was lower than the SPHERE humanitarian standard of > 75% in WaSt (58.0%) and non-WaSt (65.4%) groups [27]. The number of deaths among WaSt and non-WaSt children were not large enough to make meaningful comparison of treatment outcomes. Although children with WaSt seemed to have gained weight at a higher velocity than those without WaSt, weight velocity was below the SPHERE humanitarian standard of ≥ 4.0 g/kg/day [27]. The prevailing harsh environmental conditions and the highly infectious disease burden in Karamoja might have slowed the weight gain velocity in children in our study. More children with WaSt were discharged as non-response to treatment than those without WaSt. The default rate of 19.8% and 23.3% among children with WaSt and non-WaSt children respectively in our analysis were above the acceptable limit of < 15% by the SPHERE humanitarian standard [27]. In contrast to the sub-optimal treatment outcomes seen in our analysis, some previous studies reported high performance treatment outcomes in Ethiopia [5] and Nigeria [6]. The treatment outcomes reported in our study raise programme effectiveness concerns; which could partly be due to health system weaknesses or poor child care practices at the community level or both. Given the precarious food security situation in Karamoja; the high rate of non-response in WaSt children, treatment defaults, discharged as cured without nutritional recovery, long treatment periods coupled with low recovery rate warrant further investigations. There is a need for further research to uncover the factors influencing the sub-optimal treatment outcomes. Consideration should be given to efforts to improve OTC programme effectiveness in Karamoja.

### Incidence rate of recovery

Non-WaSt children had a slightly higher incidence rate of recovery compared to children with WaSt. Nonetheless, the median time to recovery in WaSt and non-WaSt children was not statistically different. Children with MUAC < 10.5 cm at admission had the lowest incidence of recovery which is explained by the long median time to recovery compared to those with MUAC 10.5–11.4 cm and MUAC ≥ 11.5 cm. A previous study indicates wasted children are likely to experience lower incidence rates of recovery than those with edematous malnutrition [28]. The possible explanation for the low incidence rate of recovery seen in this analysis could be because children in this cohort had SAM. Children in Kaabong district had a longer treatment duration than those in Moroto and Nakapiripirit districts. The recommended length of stay in treatment is < 60 days as per the SPHERE Humanitarian standard and the national IMAM guidelines [22, 27]. The variation in treatment outcomes in the districts might be due to differences in the quality of services and childcare practices. Efforts to investigate and address the difference in treatment outcomes in Karamoja are recommended.

### Factors associated with time to recovery

In the present study, age, low MUAC at admission and geographical location were associated with time to recovery. The probability of recovery was significantly higher in children aged 24–59 months (1.30 times), children with MUAC 10.5–11.4 cm (2.03 times); and MUAC ≥ 11.5cm (3.31 times) at admission, and in those living in Moroto (3.34 times) and Nakapiripirit (1.95 times) districts. Sex was not significantly associated with time to recovery at bivariate and multivariate analyses. However, it was retained in the model given the marked sex difference of WaSt patterns in the analysis. In the model, children without WaSt had 1.20 times higher probability of recovery compared to children with WaSt though not statistically significant. Our findings were consistent with a previous study that found children aged > 24 months had 1.25 times higher probability of recovery from SAM compared to children aged < 24 months [29]. In contrast, older children aged > 3 years were less likely to recover from SAM compared with younger children aged < 3 years in previous studies [28, 30]. Children aged < 24 months in our study probably had worse severity of acute malnutrition leading to poor treatment outcomes. From our analysis, children with very low MUAC <10.5cm, being < 24 months old and living in Kaabong district were less likely to recover from SAM. The low values of MUAC at admission seen in this study could be a consequence of poor health-seeking behaviors and child care practices. Particular attention should be given to early community case finding, referral, and treatment of SAM and WaSt to avoid poor outcomes especially among children aged less than 24 months in Karamoja. Barriers to the uptake of OTC services and remaining in treatment till recovery from SAM need to be investigated and addressed.

### Limitations

The use of secondary datasets taken from routine programmes in health facilities has inherent limitations. Random error is often associated with a fixed sample size used in retrospective studies. We obtained a ±1.68% tolerable error from our sample size of 788 using the modified Kish Leslie formula [31]. We considered 95% level of confidence, a 3% pooled prevalence of WaSt [9], and a design effect of 2. We concluded that between 47.02% to 50.38% of the children in our sample had WaSt.

The exclusion of children with incomplete data, cleaning of missing data and anthropometric measurement outliers may be potential sources of selection bias. However, we censored out of range and implausible anthropometric data. We could not censor Z-scores outliers based on the WHO flagging criteria because the dataset was a cohort of children with SAM [24]. This approach may have introduced selection bias in our analysis. Our analysis included only children who had complete data on all four anthropometric deficits; MUAC, WAZ, WHZ, and HAZ in the dataset. Selection bias may have been minimal because median age and sex distribution between children in our sample and those excluded from this analysis did not differ. Distribution of WaSt and non-WaSt had no statistical difference in the study districts. In the study setting, acute malnutrition was screened using MUAC and pitting edema per the national guidelines in the community.[22]. This may have introduced selection bias into our study. Children with normal MUAC but low WHZ may have been missed in our study. Therefore, our results should be interpreted in consideration with this limitation. Future studies should consider using data from MUAC and WHZ–based community case search programme settings.

Analysis of data on patient treatment is prone to information bias due to erroneous measurements by health workers and irregular patient attendance to OTC. Nevertheless, information bias may have been minimal due to the use of standard of operation (SOP) manuals for OTC and the technical assistance provided by CUAMM to health workers for OTC services in Karamoja. We used the OTC programme admission criteria particularly for SAM to inform eligibility of study participants [22] We also censored children who were discharged as cured but had not achieved nutritional recovery in the Cox proportional hazard regression. This might have minimized information bias due to differential misclassification of exposure given knowledge of the treatment outcome.

Our study was cross-sectional which precluded control of all possible confounding factors because of missing or inadequate measurement of variables. We were unable to control for possible confounding factors such as comorbidity because they were unavailable in the dataset; this would have explained the sub-optimal treatment outcomes and response seen in the analysis. There was no interaction between variables at the multivariate analysis. A robust standard error was used to minimize any noise in the data in the multivariate Cox proportional hazard regression analysis. The findings of this analysis could be useful baseline information for future studies and contribute evidence on the need to optimize the current OTC protocol to detect and treat WaSt cases. Future research could consider the use of data from OTC programme with WHZ ≥ -2 or MUAC ≥ 12.5 cm discharge criteria for recovery.

## Conclusions

The high prevalence of children with WaSt in this analysis uncovers a public health priority group that deserve particular attention by current therapeutic feeding programmes. The existing therapeutic feeding protocol could be used to detect and effectively treat children with WaSt. However, further research work on community case finding and referral of WaSt in therapeutic feeding programmes is recommended. Factors associated with the high rate of non-response, long duration of treatment and low recovery rates in WaSt children need to be identified and addressed for effective OTC programme in Karamoja.

## Supporting information

S1 Study dataset (XLS)

## Abbreviations

aHR: Adjusted Hazard Ratio
CI: Confidence Interval
cHR: Crude Hazard Ratio
CSB: Corn Soya Blend
CUAMM: Medici con l’Africa or Doctors with Africa
HAZ: Height-For- Age Z-scores
IMAM: Integrated Management of Acute Malnutrition
ITC: Inpatient Therapeutic Care
IQR: Interquartile Range
MAM: Moderate Acute Malnutrition
MUAC: Mid Upper Arm Circumference
MoH: Ministry of Health
ODK: Open Data Kit
OTC: Outpatient Therapeutic Care
RUTF: Ready to Use Therapeutic Foods
RUFORUM: Regional Universities Forum for Capacity Building in Agriculture
UNICEF: United Nations Children's Fund
SAM: Severe Acute Malnutrition
SFP: Supplementary Feeding Programme
WAZ: Weight -for-age Z-scores
WaSt: Wasting and stunting
WHO: World Health Organization
WHZ: Weight-for-height Z-scores
